# Our Experiences with Asparaginase Activity Measurements in Children with Lymphoblastic Diseases

**DOI:** 10.3390/children10071160

**Published:** 2023-07-02

**Authors:** Judit Müller, Petra Egyed, Daniel Erdelyi, Krisztian Kovacs, Katalin Mudra, Sandor Szabo, Balint Egyed, Kovacs Gabor

**Affiliations:** 1 2nd Department of Pediatrics, Semmelweis University, 1094 Budapest, Hungary; 2 County Hospital Fejer, Szent Gyorgy Hospital, 8000 Szekesfehervar, Hungary; 3 Department of Laboratory Medicine, Semmelweis University, 1089 Budapest, Hungary

**Keywords:** asparaginase, therapeutic drug monitoring, asparaginase enzyme activity, silent inactivation, lymphoblastic malignancy

## Abstract

Background: Asparaginase is a key component of chemotherapy protocols for the treatment of lymphoblastic malignancies among children. Adequate asparagine depletion is an important factor to achieve optimal therapeutic outcomes. Methods: Over a 3.5 year period, 106 patients were monitored for asparaginase activity (329 samples) in a single center of the Hungarian Pediatric Oncology–Hematology Group. In Hungary, three asparaginase products are available: native E. coli ASNase (Kidrolase), a pegylated form of this enzyme (Pegaspargase) and another native product from Erwinia chrysanthemi (Erwinase). A retrospective data analysis was performed. Results: In 81% (268/329) of our patients, AEA levels were in the optimal therapeutic range of over 100 IU/L. Of 106 patients, 13 (12%) were diagnosed with ‘silent inactivation’. Conclusions: Monitoring of AEA can help to identify patients with ‘silent inactivation’ and their asparaginase therapy can thus be optimized.

## 1. Introduction

Treatment of lymphoblastic malignancies among children has a high success rate, with acute lymphoblastic leukemia (ALL) having an overall survival approaching 90% and lymphoblastic lymphoma (LBL) with 80% [1,2,3]. This success is partly due to the use of an intensive combination of chemotherapeutic agents, and one of them is asparaginase (ASNase).

Healthy cells synthetize the non-essential amino acid asparagine (ASN) from aspartate (ASP) and glutamine (GLN), catalyzed by asparagine synthetase (ASNS). Lymphoblasts have no ASNS, so their growth depends on ASN in serum. ASNase converts ASN to aspartic acid and ammonia. Adequate depletion of the systemic ASN pool leads to apoptosis of lymphoblasts [4,5]. The physiological ASN level is approximately 40–80 μmol/L [6]. The treatment efficacy of ASNase is based on the sustained adequate depletion of ASN in sera. Serum ASN concentrations should be below 3 μmol/L and deamination of ASN should be over 90% [7].

Enzymes with ASNase activity can be divided into bacterial-type and plant-type enzymes [8]. Drugs used in human medicine are bacterial products [9]. Three ASNase products are available in Hungary, derived from two different bacterial sources: Escherichia coli and Erwinia chrysanthemi. Native E. coli ASNase (Kidro-lase) is a pegylated form of this enzyme (Pegaspargase), and another native product is from Erwinia chrysanthemi (Erwinase). These different ASNase products have the same mechanism of action, efficacy and side effects, but differ significantly in their pharmacokinetics. Comparative pharmacokinetic studies of different ASNase products have determined the half-life (t1/2) of ASNase enzyme activity (AEA). The serum half-life (t1/2) is 0.65 days for Erwinase, 1.28 days for Kidrolase and 5.73 days for Pegaspargase [10,11].

ASPnase is a high-molecular-weight protein, and it is not excreted renally. Proteolytic enzymes responsible for ASPnase metabolism are ubiquitously distributed in tissues, so the exact role of the liver is unknown. Inactivation of E. coli ASNase can be detected in up to 60% of cases due to anti-ASNase antibodies [12]. These antibodies rapidly neutralize circulating ASNase. This can cause clinical hypersensitivity reactions, but sometimes without any clinically evident allergic reaction, a so-called silent inactivation (SI) [13,14]. Different study groups have found different prevalences of SI in the range of 8–30% [15,16]. Premedication with antihistamines or steroids can reduce the symptoms of the allergic reaction but may not prevent antibody development [13].

AEA measurement is a good method to monitor clinical effectiveness. Therapeutic drug monitoring (TDM) is used to find patients with inadequate AEA. Complete ASN depletion can be the result of an AEA over 100 IU/L. A cut-off of ≥100 IU/L has been confirmed and used in many clinical trials [12,17,18]. An incomplete ASNase dose in ALL therapy leads to inferior outcomes both in children and adolescents compared with those who receive the majority of the intended dose of ASNase [19,20]. We have to try to optimize the service of planned ASNase therapy [21,22].

Since May 2018, AEA measurement has been available in the Department of Laboratory Medicine of Semmelweis University. In the current work, we present our experience with AEA in the light of clinical courses.

## 2. Materials and Methods

### 2.1. Study Population

The participants were children aged 1.0 to 17.9 years diagnosed with ALL and LBL and treated between May 2018 and September 2021 on the Hematological Ward of the second Department of Pediatrics in Semmelweis University, Budapest.

### 2.2. Asparaginase Treatment

ASPnase products can be given by intramuscular (im.) injection or intravenous (iv.) infusion. In Hungary, we only use the iv. method for each ASPnase product, no im. administration was performed, because im. administration is painful and can cause anxiety in children.

In the study years, the ALL-IC-BFM-2009 protocol was used for patients with de novo ALL, ALL-IC-REC-2016 was used for children with relapsed ALL and LBL-2009 and LBL-2018 were used for lymphoblastic lymphoma patients. Patients with de novo ALL were stratified into three risk groups: standard risk (SR), medium risk (MR) and high risk (HR). In the first part of the study period, Kidrolase was used during the induction phase (5000 IU/m^2^/dose iv. in a 2 h long infusion, every third day, 8 times, starting on day 12). In the re-induction phase 10,000 IU/m^2^/dose (4 times from day 7 to day 17, iv. in 2 h) was used. Since April 2021, 1500 UI/m^2^/dose Pegaspargase was administered in 2 h iv., twice at 14 days intervals during the induction phase and once in re-induction (on day 7). HR patients received 6 times 1500 UI/m^2^/dose of Pegaspargase for each HR block. LBL and relapsed ALL (REL) patients received no Kidrolase, but Pegaspargase once in each 5 day long block. Patients who developed severe clinical hypersensitivity or SI during ASPnase therapy were switched to an alternate ASPnase preparation. In the case of a Kidrolase hypersensitivity reaction or SI, Pegaspargase was administrated (1500 UI/m^2^/dose replaced 4 Kidrolase). In the case of an allergic reaction or SI for Pegaspargase and third line ASPnase treatment, Erwinase was administered in a dose of 10,000 IU/m^2^/dose every second day. According to the pharmacokinetics and t1/2, one dose of Pegaspargase was replaced with seven doses of Erwinase.

### 2.3. Asparaginse Enzyme Activity Measurement

**Figure 1:**
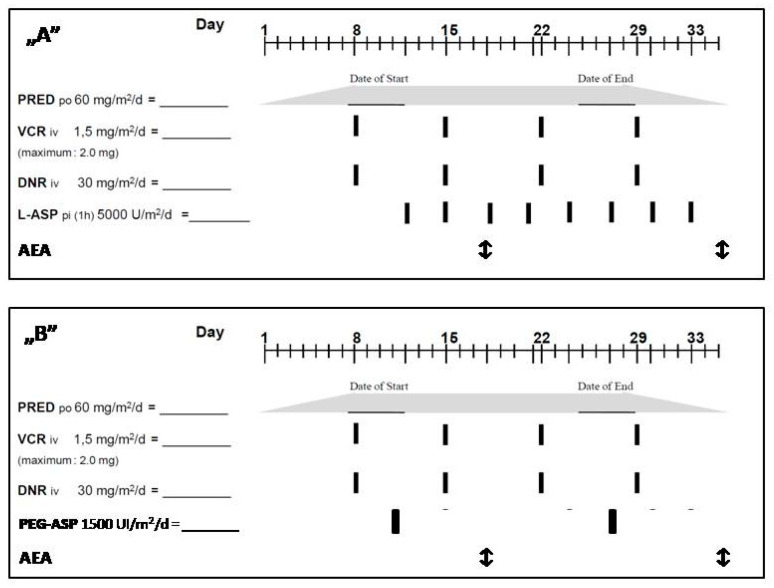
Therapeutic schema of induction chemotherapy with doses of asparaginase and time points of AEA measurements.

Blood collection for AEA measurements was performed 2–3 days after Kidrolse, 4–7 days after Pegaspargase and 2 days after Erwinase administration. K3-EDTA anticoagulated blood was taken from a central venous catheter or by peripheral vein phlebotomy (Figure 1). Samples were centrifuged (room temperature, 10 min, 3500 RPM) and after that plasma samples were frozen until testing. Asparaginase Activity Assay kits (MAK007) were purchased from MERCK (Merck kft. Budapest, Hungary) for AEA determination. ELISA assays were performed on a DAS APE ELITE ELISA instrument (MediLabKft. Szentendre). Tests were carried out according to the kit description, and Microsoft Excel 2016 was used for the required calculations. The results of AEA measurements were divided into three groups: (1) very low (0–50 IU/L), (2) low (50–100 IU/L) and (3) therapeutic level (>100 IU/L).

There was no ASP level measurement among our patients. Direct measurement of anti-ASPnase antibodies is difficult and not available in Hungary.

## 3. Results

### 3.1. Patient Characteristics

During the study period, 106 children were treated with any ASPnase product due to their lymphoid malignancy. The male/female ratio was 1.58:1 and the mean age at diagnosis was 8.0 years (13 months–17.9 years). Ninety-one children had de novo ALL, eight were treated because of REL and seven had LBL. Of the de novo ALL patients, 10 were at SR, 60 at MR and 21 were treated on the HR arm. As children with SR and MR ALL received ASP treatment with the same timings, they were taken in one group (SR + MR). In the SR + MR group, the male/female ratio was 1.59:1 and the mean age at diagnosis was 7.2 years (13 months–17.9 years). In the HR group, the male/female ratio was 1.33:1 and the mean age at diagnosis was 6.9 years (1.5–16.9 years). In the REL + LBL group, the male/female ratio was 2:1 and the mean age at diagnosis was 13.20 years (4.7–17.5 years).

### 3.2. ASPnase Monitoring

Altogether, 329 AEA level measurements were performed for 106 patients. The 70 patients in the SR+MR group had 190 AEA level measurements taken. Patients had 1–4 measurements taken and the mean AEA was 281 IU/L. The 21 HR ALL patients had 95 AEA level measurements taken. One patient had only one, but another one had altogether seven AEA measurements taken. The mean AEA level in this group was 345 IU/L. Data of 15 patients with REL or LBL were collected in one group (REL + LBL); they had 44 AEA measurements taken. These children had 1–4 measurements taken each, with a mean AEA level of 385 IU/L. Patient’s baseline characteristics and AEA levels are shown in Table 1.

**Figure 2:**
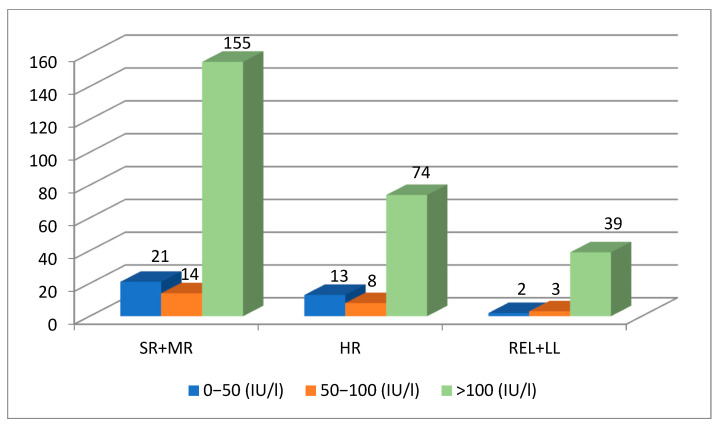
AEA level distribution for the three therapeutic groups.

**Figure 3:**
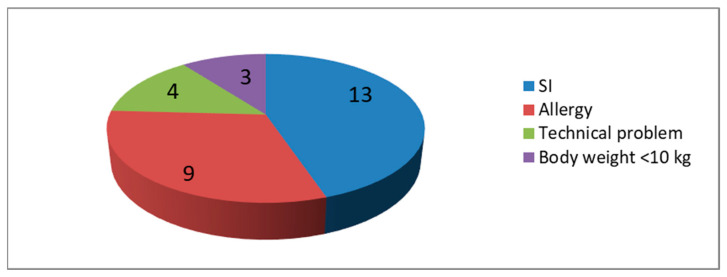
Factors behind low (0–50 IU/L) AEA in 29 patients.

In 81% of cases (268/329), the AEA level was in the optimal therapeutic range, at over 100 IU/L. There were 36 cases with AEA levels lower than 50 IU/L, 25 of these were between 50 and 100 IU/L. These 36 low AEA levels were measured in 29 children (Figure 2). In the SR + MR group, there were three boys with suboptimal AEA levels after Kidrolase administration and their body weights were below 10 kg. In four cases, a technical problem was detected as the cause of the low AEA. Nine children had ASPnase inactivation due to auto-antibody production with hypersensitive reactions. All these cases were after the induction phase. These patients were switched to another ASPnase product. In six cases, Pegaspargase was given as a second line treatment after hypersensitivity to Kidrolase. Additionally, in three cases, Erwinase was given after a Pegaspargase allergic reaction. In 13 children, SI was proven with a low AEA without any clinical signs of an allergy (Figure 3).

## 4. Discussion

The aim of this publication is to assess our experience with AEA in children with lymphoblastic malignancies in one center of the Hungarian Pediatric Oncology–Hematology Group. In this retrospective study, altogether 329 AEA levels were measured in 106 patients.

Serum ASN level monitoring is the best option to analyze ASNase therapy. However, direct measurements of ASN are very complicated and cannot be used in clinical practice. All recommendations suggest AEA monitoring to detect the real efficacy of ASNase therapy. In vitro and in vivo studies showed the clinically relevant correlation between serum ASN levels and the measured AEA. The majority of these studies and study groups recommend using AEA for clinical decisions. Anti-ASNase antibodies can indeed be measured, but in this study in Hungary we did not have the opportunity to do. Van der Sluis et al. published consensus recommendations for AEA monitoring in 2016 [17]. The Dutch Childhood Oncology Group defined SI in patients with a PEGasparaginase activity of <100 IU/L at day 7 + 1 or <20 IU/L at day 14 ± 1 after iv. administration without any clinical sign of hypersensitivity [15]. SI was defined as two consecutive activity measurements less than 100 IU/L in the DFCI ALL 00–01 study [19]. In 81% (268/329) of cases, the AEA level was in the optimal therapeutic range of over 100 IU/L. Levels between 50 and 100 IU/L do not necessarily mean the absolute inactivation of ASPnase. We only had 25 cases with an AEA between 50 and 100 IU/L. We found at least once an AEA lower than 50 IU/L in 29 children. In four cases, there were some technical problems. There were three boys with body weights lower than 10 kg, who had an AEA below 50 UI/L in the induction phase. Hepatic clearance of drugs can be higher in infants and preschool children as the liver blood flow is higher compared with adults, owing to the larger ratio of the liver to the total body mass in the former population [23,24]. Further pharmacokinetic studies can prove this theory.

In nine children, ASPnase inactivation was due to auto-antibody production with a hypersensitive reaction. These patients were switched to another ASPnase product. From 106 patients, 13 (12%) were diagnosed with SI and their ASPnase treatment was also switched. Before AEA level monitoring was available, we had no way to diagnose SI and these patients did not get the optimal dose of ASPnase. Currently, this 12% of children have also completed their ASPnase treatment and hopefully their possibility of relapse will be lower.

The aim of this work was to focus on the clinical relevance of AEA measurements. Data collection and analyses are planned to evaluate the response rate survival and MRD levels. As ASP is used in all pediatric protocols for lymphoblastic malignancies, but not generally used in adult patients, pediatric data can be relevant and informative. We began with AEA monitoring to give recommendations and experience about the importance of these measurements in the whole of Hungary (and for all pediatric cases). Our experience demonstrates how important it is to measure the AEA for the whole pediatric population with ALL.

## 5. Conclusions

Our current work proved the importance of AEA measurements. Earlier, we had no possibility of detecting SI. With the help of AEA measurements, almost 10% of patients were diagnosed with SI and their ASPnase therapy could be optimized.

Monitoring of AEA can help to identify patients with SI and to differentiate between real allergic reactions from infusion reactions. A lower toxicity and improved survival rates of pediatric ALL and LBL patients can be achieved with individualized administration of the anticancer drugs, especially ASPnase products.

## Figures and Tables

**Table 1:** Patient’s characteristics and AEA levels.

	SR + MR	HR	REL + LBL	∑
No. of patients	70	21	15	106
Male/female	1.59:1	1.33:1	2:1	1.58:1
Median age (range)	7.2 years (13 mo–17.9 y)	6.9 years (1.5 y–16.9 y)	13.2 years (4.7 y–17.5 y)	8.0 years (13 mo–17.9 y)
No. of samples	190	95	44	329
Samples (no.)/patient mean	1–4 2.7	1–7 4.5	1–5 2.9	1–7 3.4
Mean AEA (IU/L)	281	345	385	337
0–50 (IU/L)	21	13	2	36
50–100 (IU/L)	14	8	3	25
>100 (IU/L)	155	74	39	268

## Data Availability

Original data can be obtained from Judit Müller upon request.

## References

[1] Pieters R., de Groot-Kruseman H., van der Velden V., Fiocco M., Berg H.V.D., de Bont E., Egeler R.M., Hoogerbrugge P., Kaspers G., Van der Schoot E. (2016). Successful Therapy Reduction and Intensification for Childhood Acute Lymphoblastic Leukemia Based on Minimal Residual Disease Monitoring: Study ALL10 from the Dutch Childhood Oncology Group. J. Clin. Oncol..

[2] Reedijk A.M.J., Coebergh J.W.W., de Groot-Kruseman H.A., van der Sluis I.M., Kremer L.C., Karim-Kos H.E., Pieters R. (2021). Progress against childhood and adolescent acute lymphoblastic leukaemia in the Netherlands, 1990–2015. Leukemia.

[3] Burkhardt B., Hermiston M.L. (2019). Lymphoblastic lymphoma in children and adolescents: Review of current challenges and future opportunities. Br. J. Haematol..

[4] Müller H.J., Boos J. (1998). Use of L-asparaginase in childhood ALL. Crit. Rev. Oncol. Hematol..

[5] Müller H.-J., Beier R., Löning L., Blütters-Sawatzki R., Dörffel W., Maass E., Müller-Weihrich S., Scheel-Walter H.-G., Scherer F., Stahnke K. (2001). Pharmacokinetics of native *Escherichia coli* asparaginase (*Asparaginase medac*) and hypersensitivity reactions in ALL-BFM 95 reinduction treatment. Br. J. Haematol..

[6] Boos J., Werber G., Ahlke E., Schulze-Westhoff P., Nowak-Göttl U., Würthwein G., Verspohl E., Ritter J., Jürgens H. (1996). Monitoring of asparaginase activity and asparagine levels in children on different asparaginase preparations. Eur. J. Cancer.

[7] Avramis V.I., Panosyan E.H. (2005). Pharmacokinetic/pharmacodynamic relationships of asparaginase formulations: The past, the present and recommendations for the future. Clin. Pharmacokinet..

[8] Michalska K., Jaskolski M. (2006). Structural aspects of L-asparaginases, their friends and relations. Acta Biochim. Pol..

[9] van den Berg H. (2011). Asparaginase revisited. Leuk. Lymphoma.

[10] Asselin B.L. (1999). The three asparaginases. Comparative pharmacology and optimal use in childhood leukemia. Adv. Exp. Med. Biol..

[11] Asselin B.L., Whitin J.C., Coppola D.J., Rupp I.P., Sallan E.S., Cohen H.J. (1993). Comparative pharmacokinetic studies of three asparaginase preparations. J. Clin. Oncol..

[12] Pieters R., Hunger S.P., Boos J., Rizzari C., Silverman L., Baruchel A., Goekbuget N., Schrappe M., Pui C.-H. (2011). L-asparaginase treatment in acute lymphoblastic leukemia: A focus on *Erwinia* asparaginase. Cancer.

[13] van der Sluis I.M., Vrooman L.M., Pieters R., Baruchel A., Escherich G., Goulden N., Mondelaers V., Sanchez de Toledo J., Rizzari C., Silverman L.B. (2016). Consensus expert recommendations for identification and management of asparaginase hypersensitivity and silent inactivation. Haematologica.

[14] Lanvers C., Pinheiro J.P.V., Hempel G., Wuerthwein G., Boos J. (2002). Analytical validation of a microplate reader-based method for the therapeutic drug monitoring of L-asparaginase in human serum. Anal. Biochem..

[15] Tong W.H., Pieters R., Kaspers G.J.L., Loo D.M.W.M.T., Bierings M.B., Bos C.V.D., Kollen W.J.W., Hop W.C.J., Lanvers-Kaminsky C., Relling M.V. (2014). A prospective study on drug monitoring of PEGasparaginase and *Erwinia* asparaginase and asparaginase antibodies in pediatric acute lymphoblastic leukemia. Blood.

[16] Panosyan E.H., Seibel N.L., Martin-Aragon S., Gaynon P.S., Avramis I.A., Sather H., Franklin J., Nachman J., Ettinger L.J., La M. (2004). Asparaginase antibody and asparaginase activity in children with higher-risk acute lymphoblastic leukemia: Children’s Cancer Group Study CCG-1961. J. Pediatr. Hematol. Oncol..

[17] Avramis V.I., Martin-Aragon S., Avramis E.V., Asselin B.L. (2007). Pharmacoanalytical assays of *Erwinia* asparaginase (erwinase) and pharmacokinetic results in high-risk acute lymphoblastic leukemia (HR ALL) patients: Simulations of erwinase population PK-PD models. Anticancer. Res..

[18] Pinheiro J.P.V., Ahlke E., Nowak-Göttl U., Hempel G., Müller H.J., Lümkemann K., Schrappe M., Rath B., Fleischhack G., Mann G. (1999). Pharmacokinetic dose adjustment of *Erwinia* asparaginase in protocol II of the paediatric ALL/NHL-BFM treatment protocols. Br. J. Haematol..

[19] Vrooman L.M., Stevenson K.E., Supko J.G., O’Brien J., Dahlberg S.E., Asselin B.L., Athale U.H., Clavell L.A., Kelly K.M., Kutok J.L. (2013). Postinduction dexamethasone and individualized dosing of Escherichia Coli L-asparaginase each improve outcome of children and adolescents with newly diagnosed acute lymphoblastic leukemia: Results from a randomized study--Dana-Farber Cancer Institute ALL Consortium Protocol 00-01. J. Clin. Oncol..

[20] Wetzler M., Sanford B.L., Kurtzberg J., DeOliveira D., Frankel S.R., Powell B.L., Kolitz J.E., Bloomfield C.D., Larson R.A. (2007). Effective asparagine depletion with pegylated asparaginase results in improved outcomes in adult acute lymphoblastic leukemia: Cancer and Leukemia Group B Study 9511. Blood.

[21] Gupta S., Wang C., Raetz E.A., Schore R., Salzer W.L., Larsen E.C., Maloney K.W., Mattano L.A., Carroll W.L., Winick N.J. (2020). Impact of Asparaginase Discontinuation on Outcome in Childhood Acute Lymphoblastic Leukemia: A Report from the Children’s Oncology Group. J. Clin. Oncol..

[22] Højfeldt S.G., Grell K., Abrahamsson J., Lund B., Vettenranta K., Jónsson Ó.G., Frandsen T.L., Wolthers B.O., Marquart H.V.H., Vaitkeviciene G. (2021). Relapse risk following truncation of pegylated asparaginase in childhood acute lymphoblastic leukemia. Blood.

[23] Batchelor H.K., Marriott J.F. (2015). Paediatric pharmacokinetics: Key considerations. Br. J. Clin. Pharmacol..

[24] Gibbs J.P., Murray G., Risler L., Chien J.Y., Dev R., Slattery J.T. (1997). Age-dependent tetrahydrothiophenium ion formation in young children and adults receiving high-dose busulfan. Cancer Res..

